# Native RNA Purification Method for Small RNA Molecules Based on Asymmetrical Flow Field-Flow Fractionation

**DOI:** 10.3390/ph15020261

**Published:** 2022-02-21

**Authors:** Alesia A. Levanova, Mirka Lampi, Kiira Kalke, Veijo Hukkanen, Minna M. Poranen, Katri Eskelin

**Affiliations:** 1 Molecular and Integrative Biosciences Research Programme, Biological and Environmental Sciences, University of Helsinki, Viikinkaari 9, FI-00014 Helsinki, Finland; alesia.levanova@helsinki.fi (A.A.L.); mirka.lampi@helsinki.fi (M.L.); 2 Institute of Biomedicine, University of Turku, FI-20014 Turku, Finland; kiira.m.kalke@utu.fi (K.K.); veijo.hukkanen@utu.fi (V.H.)

**Keywords:** AF4, antiviral siRNA, small RNA purification

## Abstract

RNA molecules provide promising new possibilities for the prevention and treatment of viral infections and diseases. The rapid development of RNA biology and medicine requires advanced methods for the purification of RNA molecules, which allow fast and efficient RNA processing, preferably under non-denaturing conditions. Asymmetrical flow field-flow fractionation (AF4) enables gentle separation and purification of macromolecules based on their diffusion coefficients. The aim of the study was to develop an AF4 method for efficient purification of enzymatically produced antiviral small interfering (si)RNA molecules and to evaluate the overall potential of AF4 in the separation of short single-stranded (ss) and double-stranded (ds) RNA molecules. We show that AF4 separates monomeric ssRNA from dsRNA molecules of the same size and monomeric ssRNA from multimeric forms of the same ssRNA. The developed AF4 method enabled the separation of enzymatically produced 27-nt siRNAs from partially digested substrate dsRNA, which is potentially toxic for mammalian cells. The recovery of AF4-purified enzymatically produced siRNA molecules was about 70%, which is about 20% higher than obtained using anion-exchange chromatography. The AF4-purified siRNAs were not toxic for mammalian cells and fully retained their biological activity as confirmed by efficient inhibition of herpes simplex virus 1 replication in cell culture. Our work is the first to develop AF4 methods for the separation of short RNA molecules.

## 1. Introduction

Small RNA (sRNA) molecules (<200 nucleotides, nt) that do not code for any proteins, but rather possess regulatory functions, have been found in cellular organisms representing all domains of life, and are also encoded by some viruses [1,2,3,4,5]. Despite many sRNAs having already been discovered and characterized both structurally and functionally, new classes of sRNA, specific for a particular organism, tissue, or (patho)physiological conditions are still to be described [6]. Among the most abundant non-coding RNAs are ribosomal and transfer RNAs, which are engaged in protein synthesis; small nuclear RNAs, mostly involved in splicing of messenger (m)RNAs; and small nucleolar RNAs, which function in chemical modifications of various classes of RNAs [7]. MicroRNAs (miRNAs) and small interfering (si)RNAs are double-stranded (ds) non-coding RNAs with two-base 3′-terminal overhangs processed from longer dsRNA molecules by Dicer [8]. The size of siRNAs and miRNAs depends on the organism and is typically in the range of 21–27 nt [9,10,11]. These short dsRNAs have a significant role in cell physiology and multiple disease pathogenesis (see, for example, [12,13,14,15,16]). The mechanism of siRNAs and miRNAs action is based on their complementary pairing with a target mRNA resulting in translation inhibition and mRNA degradation, a phenomenon called RNA interference (RNAi). 

RNAi in mammalian cells can be triggered by the delivery of exogenous siRNAs into the cell. This method has become a powerful tool for the analysis of protein functions and for drug discovery. SiRNA drugs became a therapeutic reality in 2018, when Onpattro (patisiran) was launched for the treatment of hereditary transthyretin-mediated amyloidosis. It was quickly followed by Givlaari (givosiran) for the control of acute hepatic porphyria and Oxlumo (lumasiran) for the treatment of primary hyperoxaluria type 1. In addition to genetic diseases, siRNA-based therapies are being actively developed to control viral infections [17] and cancer [18]. Furthermore, antisense therapy based on small ssRNA molecules has proven to be a powerful approach in drug development with ten antisense oligonucleotide drugs approved since 1998 [19], and oncolytic viruses have been constructed expressing antisense elements to oncogenes [20]. Other classes of therapeutic small RNA molecules under research and development include RNA aptamers, small activating dsRNAs, ribozymes, riboswitches, and synthetic guide RNAs [19]. Furthermore, the immunostimulatory activity of RNAs has been recognized as a possible means to develop more potent RNA-based therapeutics and vaccine adjuvants [21,22].

Short ssRNA oligonucleotides for research or medical use can be synthesized by solid-phase phosphoramidite synthesis [23], while longer ssRNAs are traditionally produced from DNA templates using DNA-dependent RNA polymerase of T7, T3, or SP6 bacteriophage [24,25,26,27,28]. Post-synthetic annealing of complementary synthetic RNA oligonucleotides or enzymatically produced ssRNAs is routinely used to prepare siRNA and dsRNA molecules, respectively. An alternative approach is to use RNA-dependent RNA polymerase from bacteriophage phi6 for enzymatic production of high-quality dsRNA from ssRNA template [17,29,30]. The resulting dsRNA can be subsequently processed with the Dicer enzyme to obtain a swarm of siRNAs [17,31,32]. Depending on the synthesis process and the end-product, the generated RNA molecules must be purified from aberrant sequences, traces of template ssRNA, undigested or partially digested dsRNAs, enzymes used for synthesis, and nucleoside triphosphates (NTPs). Purification of ssRNAs is typically more challenging than dsRNAs, and elevated temperature or denaturing agents (e.g., urea) are used to prevent the formation of stable secondary and tertiary structures and enhance separation [33,34,35,36,37]. The conventional approach to purify nucleic acid molecules is denaturing urea-polyacrylamide gel electrophoresis (urea-PAGE) followed by extraction of desired molecules from the gel [38,39]. Although the method is reliable and provides high resolution and purity of specific RNA molecules, it is laborious, lengthy, difficult to scale up, and gives a low RNA yield (about 10%) with acrylamide contaminants [37]. Furthermore, distinctive three-dimensional architectures of RNAs are critical for their biological functions, and denaturation of RNA molecules should be avoided since renaturation to biologically active conformations is a non-trivial task [40].

To overcome limitations of urea-PAGE, chromatographic purification based on affinity-, size-exclusion-(SEC), (ion-pair) reversed-phase-[(IP)-RP], and anion-exchange chromatography (AEX), have been extensively developed [37,41,42]. Affinity chromatography techniques for RNA purification [43,44,45], other than polyadenylated mRNAs, are complicated by the necessity to design and clone a specific tag to RNA, introducing an additional step for tag cleavage and need to prepare custom affinity resins. SEC is a gentle method that maintains RNA tertiary structures [46,47]. However, it is lengthy and typically requires pre-purification steps to remove proteins from transcription mixtures. The most common approach for chromatographic purification of synthetic ss- and ds-oligonucleotides employs reversed-phase techniques or AEX. IP-RP chromatography using a non-denaturing mobile phase has been used to separate hybridized siRNA duplexes from ssRNA impurities and truncated duplexes [48]. We have previously developed a method for the purification of enzymatically produced siRNA molecules using AEX on monolithic columns under native conditions at ambient temperature [49]. However, before therapeutic applications, siRNAs purified with this method must be subjected to buffer exchange, which increases total processing time and reduces siRNA yields. Consequently, alternative advanced native RNA purification methods applicable to a wide range of ds- and ssRNA molecules are still in demand. 

Field-flow fractionation (FFF) is a versatile separation technique, where separation occurs in a thin channel without a stationary phase. In asymmetrical flow FFF (AFFFF or AF4) a carrier liquid with analytes is pumped through a separation channel with a semi-permeable bottom part. The main incoming flow splits into longitudinal channel flow, which is going to the detector, and the cross-flow, which is perpendicular to the main channel flow, pushing the sample components towards the semi-permeable accumulation wall. Sample components diffuse against the cross-flow force depending on their diffusion coefficients and, hence, small sample components diffuse farther from the accumulation wall than do large particles with small diffusion coefficients. Therefore, smaller particles diffuse closer to the center of the separation channel, where the velocity of the channel flow is maximal, and elute first. In AF4, separation is based solely on the hydrodynamic sizes and is potentially achievable in any mobile phase. Previously, AF4 has been used to separate ss- and dsDNA molecules of different sizes [50,51,52] and free ssDNA from protein-bound ones [53]. The technique has also been used to separate and quantify *Escherichia coli* ribosomal and transfer RNAs [54,55]. Furthermore, AF4 has been used to characterize the size distribution of lipid nanoparticles loaded with siRNAs [56,57] and separate various miRNA carriers in the serum [58]. In these studies, however, the focus has been on the particles and the carriers and not on naked si- or miRNAs.

In the present work, we addressed the separation and purification of naked sRNA molecules using AF4. We optimized the AF4 conditions, including flow rates and buffer composition, to achieve separation and purification of enzymatically produced ss- and dsRNAs as well as siRNAs. The 88-nt-long ssRNAs and dsRNAs were separated well from each other with the developed AF4 method. Longer ssRNA molecules of 108 nt were prone to form intermolecular complexes, which could be separated from the monomeric form under non-denaturing conditions. Using AF4, enzymatically produced siRNAs were efficiently purified from partially digested dsRNA substrate. The quality of the AF4 purified enzymatically produced siRNA molecules was verified by evaluating the toxicity and biological activity of the resulting siRNAs in cell culture experiments. This demonstrated that siRNAs purified in buffered mobile phase containing a small amount of salt (20 mM NaCl) did not contain toxic, longer dsRNA, and fully retained biological activity. 

## 2. Results

**Figure 1:**
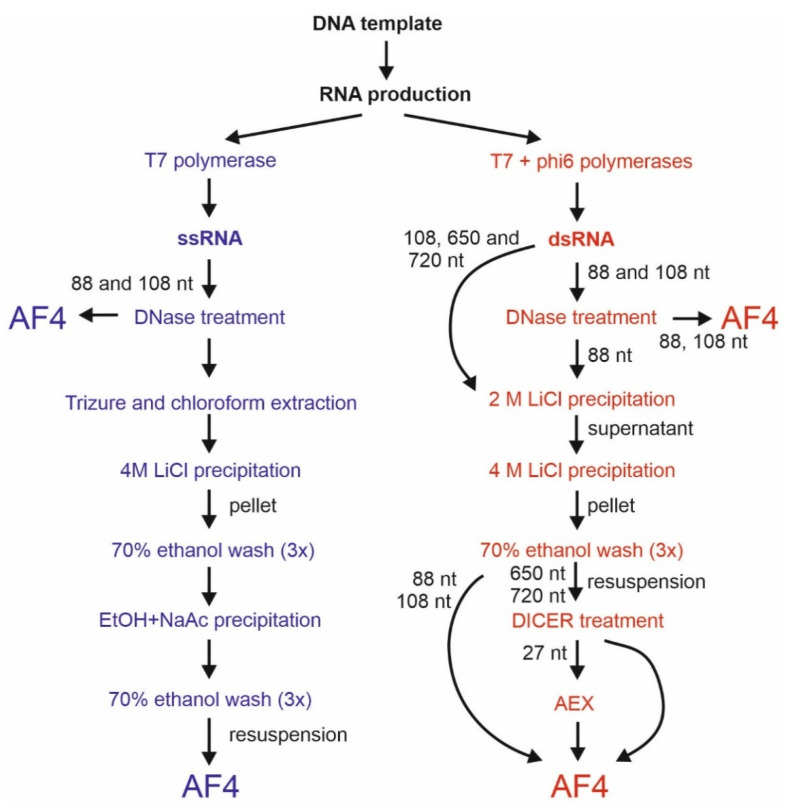
Sample preparation workflow. The ssRNA molecules (blue) were produced using T7 DNA-dependent RNA-polymerase. The dsRNA molecules were synthesized in coupled reactions that contained the T7 polymerase and phi6 RNA-dependent RNA polymerase. In addition to the DNAse I treatment, pre-purification of the ssRNA reaction mixtures included phenol–chloroform extraction followed by 4 M LiCl and ethanol precipitations. Pre-purification of dsRNA molecules (red) contained two subsequent LiCl precipitations. The 88-nt dsRNA was treated with DNase I prior to LiCl precipitation or direct purification with the asymmetrical flow field-flow fractionation (AF4). The UL29 and GFP siRNA swarms were generated from LiCl-precipitated 650-nt and 720-nt long dsRNA molecules, respectively, with Dicer and further purified with either AF4 or anion-exchange chromatography (AEX).

We studied five types of enzymatically produced sRNA molecules for their elution behavior and purification with AF4: 88-nt and 108-nt-long ssRNAs, 88-nt and 108-nt long dsRNAs, and 27-nt-long siRNAs derived from the *UL29* gene of herpes simplex virus 1 (HSV-1), enhanced green fluorescent protein (*eGFP*) gene, or bacteriophage phi6 genome (Table 1; Figure 1; see Section 4.1.1 for details). DNAse-treated ssRNA and dsRNA synthesis reaction mixtures were directly used for AF4 injections, or the samples were pre-purified before the AF4 analysis (Figure 1; see also Section 4.1.1). The short dsRNAs studied here are strong immunostimulators in human monocyte-derived dendritic cells, macrophages, glioma, and epithelial cells [31,59,60]. Furthermore, the UL29 siRNAs show antiviral activity in HSV-1 infected cell cultures and promising results have been obtained in mouse studies [31,61,62,63]. 

The folding of ssRNA affects its hydrodynamic radius and, thus, elution behavior in AF4. The biochemical and physical properties of the produced sRNAs, including parameters related to the predicted ssRNA structures [Figure S1 (88-nt RNA) and Figure S2 (108-nt RNA), Supplementary Materials], are described in Table 1.

### 2.1. AF4 Separation of Short ssRNA and dsRNA Molecules

#### 2.1.1. AF4 Set-Up for the Separation of sRNAs

In our experiments, we used two AF4 instruments currently available on the market, AF2000 MT (Postnova Analytics) and Eclipse NEON (Wyatt Technology). The channel widths were 350 µm and 400 µm, respectively. The mobile phase was 20 mM NaCl, 50 mM Tris-HCl (pH 8.0), unless otherwise indicated. The AF4 methods developed for the RNA separation with AF2000 MT and Eclipse NEON are presented in Tables S1 and S2 (Supplementary Materials), respectively.

The membrane chemistry and pore size at the accumulation wall determine the dimensions of sample components that retain in the AF4 channel for separation. The regenerated cellulose (RC) membrane was a natural choice since its isoelectric point is around 3.4 and, hence, it is negatively charged at higher pH values [66] generating repulsive forces between the membrane and negatively charged RNA molecules in the mobile phase with pH 7.0–8.0. 

Previous AF4 studies on protein–DNA aptamer interactions demonstrated that folded and non-folded poly(A) oligomers shorter than ~30 nt were poorly retained when ultrafiltration membranes with molecular weight cut-off (MWCO) of 5 or 10 kDa were used at the accumulation wall [67]. Therefore, initially, we chose to use RC membrane with a 1 kDa MWCO to maximize the retention and thus recovery of sRNA molecules. We found that with the channel-flow rate of 0.5 mL/min, the maximal applicable cross-flow rate was approximately 2.3 mL/min, which was significantly lower than the cross-flow rate routinely achieved with a typical 10 kDa RC membrane (3.5 mL/min and larger). With the Eclipse NEON system, we used 10 kDa RC membrane allowing the use of 4 mL/mL cross-flow velocity (Table S2). With this system, we could record multi-angle light scattering (MALS) and concentration data [refractive index (RI) and UV] enabling deduction of molecular weight (M_w_) for separated sample components. 

#### 2.1.2. Elution of 88- and 108-nt ssRNA Molecules 

**Figure 2:**
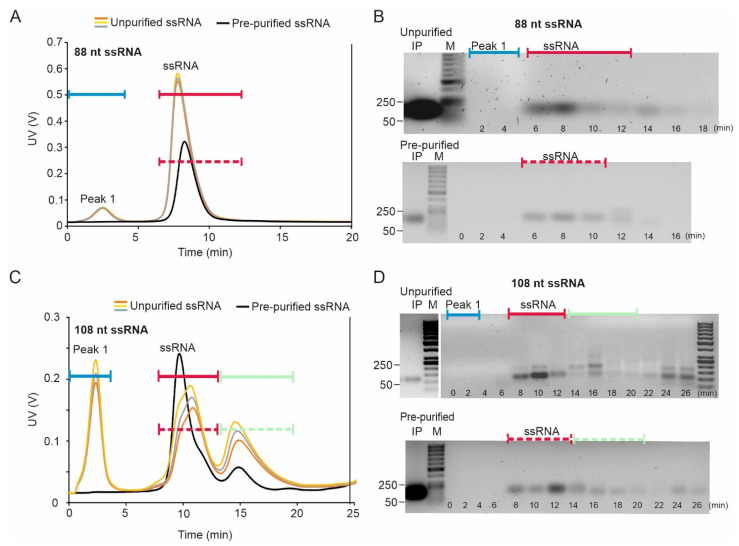
AF4 fractionation of 88- and 108-nt ssRNA molecules. (**A**) Fractogram of 88-nt ssRNA sample and (**B**) native agarose gel analysis [1% (*w*/*v*)] of the AF4 fractions from 88-nt ssRNA purification, (**C**) fractogram of 108-nt ssRNA sample, and (**D**) native agarose gel analysis [1% (*w*/*v*)] of the AF4 fractions from 108-nt ssRNA purification. Representative AF4 fractogram for the pre-purified samples (see Figure 1) is shown with black line and three replicates for the crude reaction mixtures with colored lines. Time axis shows the UV fractogram from the beginning of the elution program excluding the focusing time. UV detector response at 260 nm is given in volts (V) (left y-axis). RNA molecules were separated using AF2000 MT (Postnova Analytics) at constant cross flow of 2.3 mL/min in a mobile phase containing 20 mM NaCl, 50 mM Tris-HCl (pH 8.0), channel width of 350 µm and RC membrane with MWCO of 1 kDa. Each fraction was collected for 2 min (0.6 mL), elution start time of the fraction is marked at the bottom of the gel. Corresponding peak position in the fractogram and the gel is indicated with colored lines. The elution time of the fractions is indicated below the gels. Fraction samples loaded on the gel contained up to 300 ng RNA. M is GeneRuler 50 bp DNA ladder (Thermo Fisher). Mobility of selected dsDNA molecules (in base pairs, bp) is indicated on the left. IP is the input sample. IP loading amounts are not comparable between different panels.

The purified 88-nt ssRNAs eluted as a single peak, and the agarose gel analysis of the peak fractions showed a uniformly migrating RNA species (Figure 2A,B). The average mass recovery of the 88-nt ssRNA in the peak fraction was 85 ± 2.5%. The homogeneity and monomeric nature of the eluting ssRNA were confirmed by collecting online light scattering data using the Eclipse NEON multidetector setup that showed M_w_ of 35.0 kDa ± 0.7% throughout the peak (Figure S3A, Supplementary Materials). The difference between the root mean square radii of gyration (R_g_) for the two predicted conformers of the 88-nt ssRNA was too small (Table 1) for the molecules to be separated with AF4. 

The 108-nt ssRNA species eluted as two peaks (Figure 2C, Figures S3B and S4A, Supplementary Materials), which suggested conformational and/or size heterogeneity in the sample. Secondary structure predictions indicated that the 108-nt ssRNA molecule could potentially form more folds than the 88-nt molecule (Figures S1 and S2), and that the different isoforms have relatively large size differences (R_g_) (Figures S1 and S2; Table 1). Sample components of the early-eluting fractions of the pre-purified 108-nt ssRNA sample migrated predominantly as a single band in the agarose gel (Figure 2D). However, multiple bands were observed in the later eluting fractions. Based on the mobilities in agarose gel, we speculate that these larger RNA species are formed from intermolecular interactions between ssRNA molecules. Accordingly, the MALS data collected during AF4 separation showed that the M_w_ for the RNA in the second peak, 87.9 kDa ± 0.2%, was comparable with that of the purified dsRNA molecule, 85.1 kDa ± 0.1% (Figure S3B). This rules out the possibility that the observed second peak would represent different conformers of the monomeric ssRNA molecule. The single-stranded nature of the RNA was further verified by RNase sensitivity test (Figure S4C) confirming that the molecules in the second peak are multimers of ssRNA molecules and not dsRNA. Thus, AF4 could be used to fractionate monomers from the different oligomeric forms of the 108-nt ssRNA. The average mass recovery of the pre-purified 108-nt ssRNA in the first peak fraction was 83 ± 1.6%.

An additional peak (peak 1; Figure 2A,C) was observed in the fractograms of the crude reaction mixture (T7 ssRNA synthesis reaction mixtures), but no RNA was observed in the corresponding fractions by agarose gel electrophoresis analysis (Figure 2B,D). The position of this peak corresponds to the position in which NTPs elute (see Section 2.2.2 and Figure S6B). Importantly, no NTP peak was observed when ultrafiltration membrane with MWCO of 10 kDa was used (Figure S4) since only a tiny fraction of NTP can be retained under these conditions (see Section 2.2.2). Otherwise, the fractograms of the purified and unpurified ssRNAs were similar (Figure 2). 

#### 2.1.3. Mobile Phase Composition Affects ssRNA Elution

Cations present in the mobile phase have a strong impact on RNA folding, intermolecular interactions as well as on the interactions of RNAs with the ultrafiltration membrane and, thus, potentially affect the elution of ssRNA molecules [68,69]. Therefore, we compared behavior of the 108-nt ssRNA in two mobile phases: (i) in 20 mM NaCl, 50 mM Tris-HCl (pH 8.0) and (ii) in 10 mM Tris-HCl (pH 8.0). The lower ionic strength of the mobile phase accelerated the elution of ssRNA molecules. In addition, the peak was broader and had an extensive tail (Figure S5A, Supplementary Materials). The intensity and number of the slowly migrating higher molecular weight bands in agarose gel were reduced when AF4 fractionation was carried out using mobile phase without NaCl compared to the standard conditions containing 20 mM NaCl (Figure S5B,C). The negative charge of RNA in a buffer with 20 mM NaCl is at least partially shielded and, hence, repulsive interactions are reduced [69]. At the same time, short-range interactions, such as hydrogen bonding, Van-der-Waals, or hydrophobic forces between molecules and RC membrane at the accumulation wall are probably promoted, which translates into enhanced retention and formation of RNA intermolecular complexes. Base pairs formed on ssRNA are not stable at low ionic strength, and ssRNAs form flexible polymers with different conformations [70], which together with the enhanced interactions with RC membrane may explain the peak broadening for ssRNAs. In summary, proper optimization of the mobile phase composition can improve separation and increase the yield of ssRNAs. 

#### 2.1.4. AF4 Provides Good Separation between Small ssRNA and dsRNA Molecules in Native Conditions

**Figure 3:**
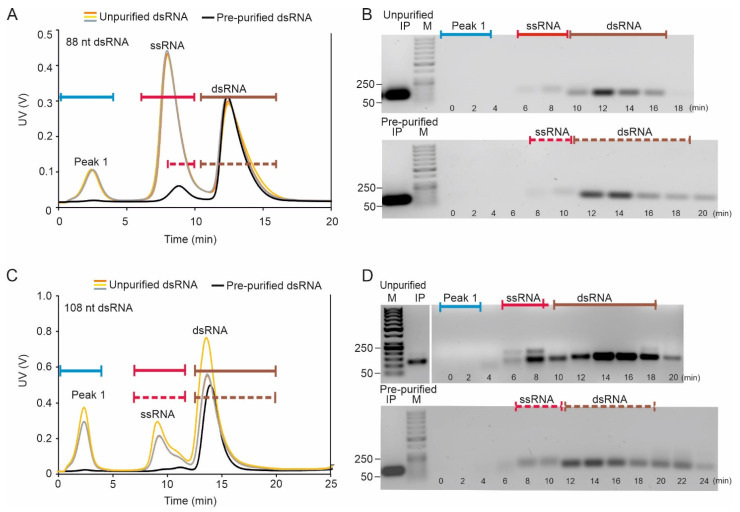
AF4 fractionation of 88- and 108-nt dsRNA molecules. (**A**) Fractogram of 88-nt dsRNA sample and (**B**) native agarose gel analysis [1% (*w*/*v*)] of the AF4 fractions from 88-nt dsRNA purification, (**C**) fractogram of 108-nt dsRNA sample, and (**D**) native agarose gel analysis [1% (*w*/*v*)] of the AF4 fractions from 108-nt dsRNA purification. Representative AF4 fractogram for the pre-purified samples (see Figure 1) is shown with black line and three replicates for the crude reaction mixtures with colored lines. Time axis shows the UV fractogram from the beginning of elution program excluding the focusing time. UV detector response at 260 nm is given in volts (V) (left y-axis). The RNA molecules were separated using AF2000 MT (Postnova Analytics) in 20 mM NaCl, 50 mM Tris-HCl (pH 8.0), at constant cross-flow of 2.3 mL/min, channel width of 350 µm and RC membrane with MWCO of 1 kDa. Corresponding peak position in the fractogram and the gel is indicated with colored lines. Each fraction was collected for 2 min (0.6 mL), elution start time of the fraction is marked at the bottom of the gel. Fraction samples loaded on the gel contained up to 300 ng RNA. M is GeneRuler 50 bp DNA ladder (Thermo Fisher). IP is the input sample. IP loading amounts are not comparable between different panels. Mobility of selected dsDNA molecules (in base pairs, bp) is indicated on the left.

AF4 fractionation of the 88-nt and 108-nt dsRNA species resulted in similar fractograms: one or two peaks were observed for the pre-purified molecules and three for the unpurified dsRNA samples. In the AF2000 MT instrument, the 88-nt dsRNA eluted between 11 and 15 min, and the 108-nt dsRNA between 12.5 and 16.5 min (Figure 3A,C). In addition, the pre-purified dsRNA samples included a minor low-intensity peak, which most probably originated from ssRNA molecules incompletely precipitated with 2M LiCl, since the retention times correspond to those observed for ssRNA species (Figure 2A–D). These ssRNA contaminates were not observed by agarose gel electrophoresis analysis of the initial input sample which emphasizes the potential of AF4 for RNA quality control. The observation also indicates that AF4 fractionation can improve the separation of ss- and dsRNA molecules compared to the conventional LiCl precipitation method. The average mass recovery of AF4 purified 88-nt dsRNA was 66 ± 2.1% of the input pre-purified sample. The average mass recovery of AF4 purified 108-nt dsRNA was 78 ± 3.8% of the input pre-purified sample, respectively.

The AF4 fractogram of the crude reaction mixture displayed a larger ssRNA peak than observed for the pre-purified sample and an early peak at ~2 min (Figure 3A,C), at the position where NTPs elute (see Section 2.2.2; Figure S6B, Supplementary Materials). Agarose gel analysis revealed that the content of the dsRNA peak is homogenous in respect of RNA size. This was supported with the M_w_ distribution data from the MALS measurements (Figure S3.) However, considering the elution behavior of the 108-nt ssRNA multimers (Figure 2C,D), there is a possibility that the 108-nt dsRNA fraction (Figure 3C) contains traces of ssRNA, although such ssRNA is barely detected by the agarose gel analysis (Figure 3D). This observation underlines the importance of analyzing all the sub-components of the analyte separately.

### 2.2. Purification of siRNA Molecules with AF4

#### 2.2.1. AF4 Separates siRNAs and Partially Digested Dicer Reaction Products

**Figure 4:**
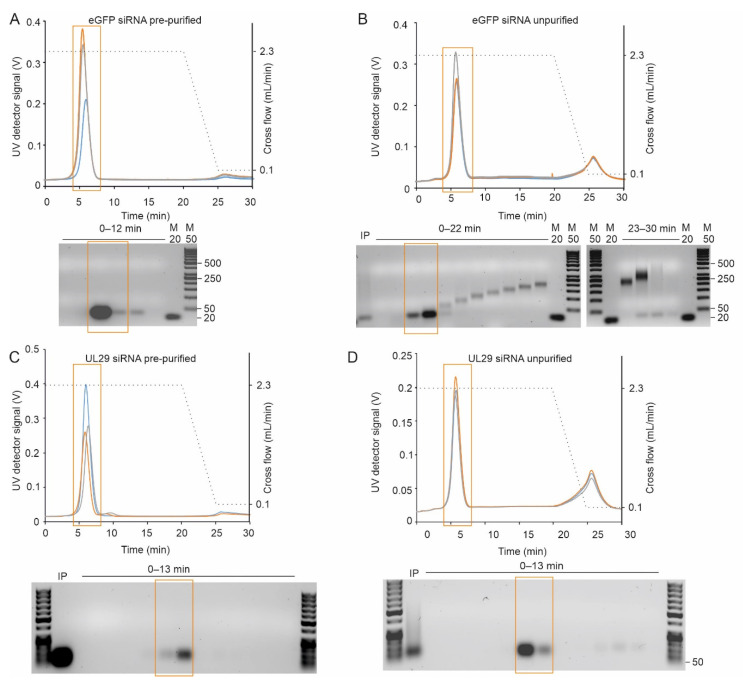
AF4 separation of siRNA molecules using AF2000 MT (Postnova Analytics) instrument. Three representative fractograms for the (**A**) pre-purified eGFP siRNA, (**B**) unpurified eGFP siRNA, (**C**) pre-purified UL29 siRNA, and (**D**) unpurified UL29 siRNA are shown with different colors. Cross-flow gradients are shown with black dashed lines (right *y*-axis). Time axis shows the UV fractogram from the beginning of elution program excluding the focusing time. UV detector response at 260 nm is given in volts (V) (left *y*-axis). SiRNA molecules were separated in a 20 mM NaCl, 50 mM Tris-HCl (pH 8.0) using constant cross-flow of 2.3 mL/min, channel width of 350 µm and RC membrane with MWCO of 1 kDa. Fractions were collected for 2 min (1 mL) from eGFP siRNA injections, and 1 min (0.5 mL) from UL29 siRNA injections. The collected fractions were analyzed in 2% (*w*/*v*; eGFP) or 1% (*w*/*v*; UL29) native agarose gels shown below the fractograms. For pre-purified eGFP, two fractions (2 mL) were pooled for gel analysis. IP refers to the input sample. M50 is GeneRuler 50 bp DNA ladder (Thermo Fisher) and M20—a No Limit 20 bp DNA fragment (Fermentas). Mobility of selected dsDNA molecules (in bp) is indicated on the right. Corresponding peak positions in the fractogram and the gels are indicated with boxes.

SiRNA molecules pre-purified with AEX eluted as one major peak, which was clearly distinct from the void volume, thus enabling the proper separation of the siRNAs from the potential non-retained sample components (Figure 4A,C). In contrast to the fractograms of the pre-purified siRNA molecules, the UV signal for the unpurified siRNA preparation produced by Dicer digestion from long dsRNA did not reach baseline level after the main peak eluted (compare Figure 4A–D). Agarose gel analysis revealed that these late-eluting fractions contained multimers of siRNA molecules (Figure 4B,D). All samples had components that eluted with zero cross-flow force, indicating a large molecule size. Such components were more abundant in the non-purified samples and might include remnants of dsRNA substrate that were not completely processed (Figure 4). 

**Figure 5:**
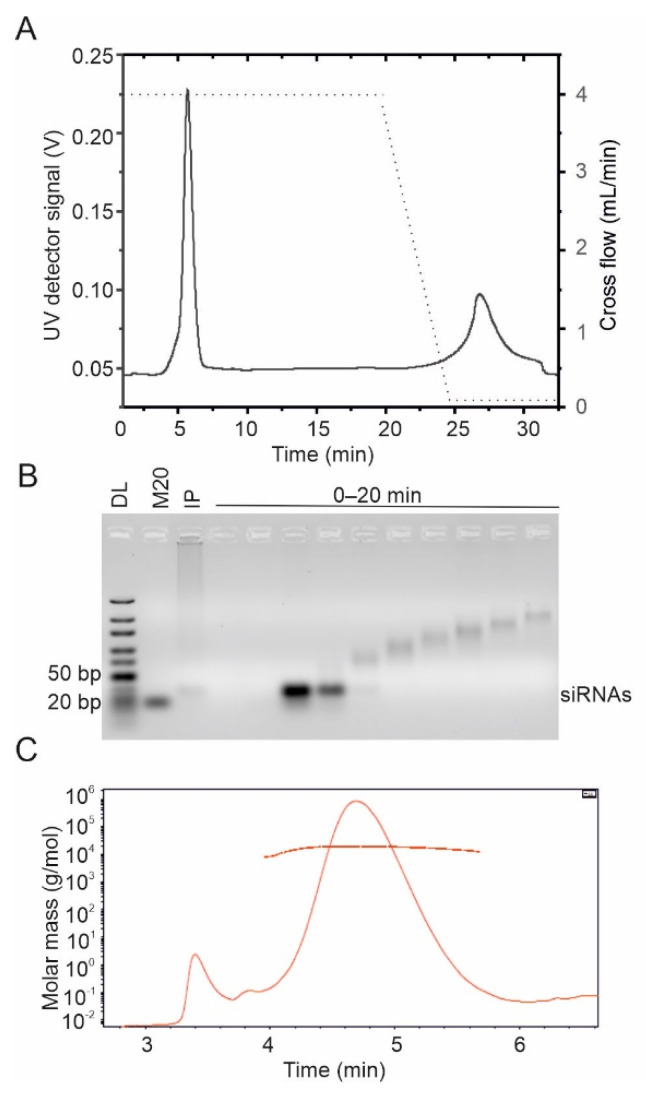
AF4 purification of siRNA molecules using Eclipse NEON multidetector system (Wyatt). A 10 kDa membrane, 400 µm channel height, and 4 mL/min cross-flow velocity were used. (**A**) A representative fractogram for the AF4 purification of siRNA swarm obtained after digestion of phi6 dsRNA with Giardia Dicer (black line). Cross-flow gradient from 4 mL/min to 0.1 mL/min is shown with black dashed line (right *y*-axis). UV detector response at 260 nm is shown in volts (V) (left *y*-axis). Time axis shows the UV fractogram from the beginning of elution program excluding the focusing time. (**B**) The fractions collected for 2 min (1 mL) from the elution step were analyzed in 4% (*w*/*v*) Nusieve CTG agarose gel. IP refers to the input sample. DL is Ultra low range DNA ladder and M20 is a No Limit 20 bp DNA fragment (Fermentas). Mobility of the selected dsDNA molecules (in bp) is indicated on the left. (**C**) Molar mass of siRNAs was calculated from light scattering and concentration data with ASTRA 8.0 software. The sample peak obtained with dRI detector is presented. The molar mass distribution is shown with a horizontal line.

In addition to the Postnova AF2000 instrument (Figure 4), the Wyatt Eclipse FFF NEON system coupled to the multi-detection platform was applied to purify siRNA molecules (Figure 5). The MALS data suggested that the molecular weight of the purified siRNAs was 17.7 kDa (Figure 5C), which was in good agreement with the theoretically calculated value of 17.3 kDa (Table 1). The derived polydispersity index (M_w_/M_n_) of 1.02 ± 2.6% confirmed the homogeneity of the sample. 

To estimate the recovery of siRNAs with a 10 kDa RC membrane, we injected known amounts (7.5–15 µg) of siRNAs pre-purified with AEX to AF4 and collected siRNA-containing fractions. After precipitation (see Section 4.1.4.), the mass recovery of the siRNA molecules was 72 ± 16%. A similar estimate for recovery, 70 ± 15%, was calculated by ASTRA 8.0 algorithm based on the collected signals from the concentration detectors (UV and RI). Thus, a 10 kDa RC membrane provided good recovery of the loaded siRNA together with the possibility to use higher flow rates without causing overpressure. 

#### 2.2.2. AF4 Fractionation Separates siRNAs from Contaminating Reaction Components

In addition to incompletely digested dsRNA molecules, which are separated from siRNAs with the developed AF4 method (Figure 4 and Figure 5), reactions contain the Dicer enzyme and potential traces of bacteriophage polymerases and NTPs, although the last two are mostly removed from dsRNA sample during LiCl precipitation step [71]. To check that proteins and NTPs do not co-elute with the siRNA fractions during AF4 separation, we mixed pre-purified siRNA molecules with NTPs or a model protein, bovine serum albumin (BSA), and separated the mixtures using Eclipse NEON multidetector system (Wyatt) under the conditions described above (see Section 2.2.1, Figure 5). BSA with a molecular weight of 66.5 kDa was well separated from siRNA peak eluting substantially later than siRNAs (Figure S6A). Bacteriophage polymerases and Giardia Dicer are all negatively charged at pH 8.0 and larger than BSA (phi6 polymerase—75 kDa, pI = 6.57; T7 polymerase—99 kDa, pI = 6.77; and Giardia Dicer—82 kDa, pI = 5.98), which should result in even longer retention in the channel and, hence, better separation from siRNAs compared to that observed with BSA. Most of the injected NTPs are removed with the cross-flow through a 10 kDa RC membrane. The fraction of NTPs (about 0.3%), which is retained in the channel, elute in the void volume, which is distinct from the siRNA peak, when 20 mM NaCl, 50 mM Tris-HCl (pH 8.0) is used as mobile phase (Figure S6B). 

#### 2.2.3. Mobile Phase Influences Elution and siRNA Purification by AF4

**Figure 6:**
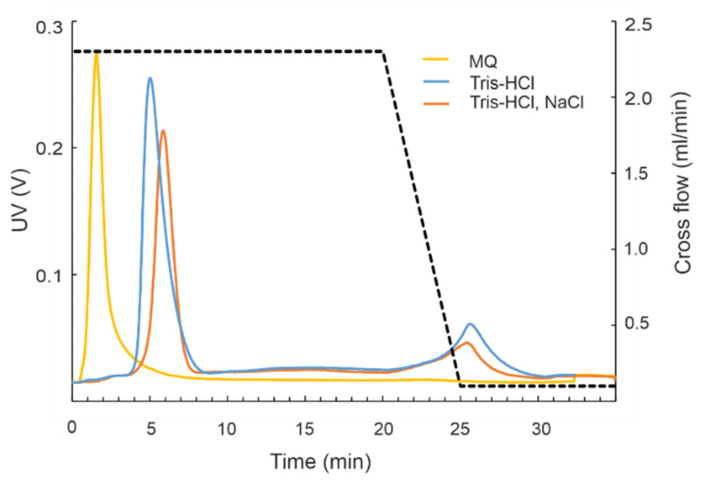
Effect of mobile phase composition on the elution of siRNA molecules in AF4. Overlaid fractograms from experiments performed with AF2000 MT instrument. Three mobile phases were used: 20 mM NaCl, 50 mM Tris-HCl pH 8.0 (orange line); 10 mM Tris-HCl pH 8.0 (blue line); or plain water (yellow line). The Dicer-processed eGFP dsRNA molecules were injected in 100–150 μL. Injection loop volume was 500 μL. Time axis shows the UV fractogram from the beginning of elution program excluding the focusing time. Cross-flow gradient is shown with dashed line. UV detector response at 260 nm is given in volts (V) (left *y*-axis).

Preparation of siRNAs for therapeutic applications would benefit from the decreased number of processing steps and reduced hands-on time. Purification of siRNAs using water as a mobile phase during AF4 would eliminate the need for a desalting procedure and potentiate higher siRNA yield. We evaluated the separation of siRNAs in Tris buffer that did not contain NaCl as well as in ultrapure water (Figure 6). A fractogram from AF4 experiments with the standard mobile phase of 20 mM NaCl, 50 mM Tris-HCl showed a single distinct siRNA peak that eluted after ~6 min. When the analysis was performed in 10 mM Tris-HCl buffer lacking NaCl, the retention time slightly decreased and the peak was broader. When the fractionation was carried out in ultrapure water, the siRNAs eluted as high-intensity peaks at the beginning of elution in the void volume along with the non-retained small sample components. Furthermore, under these conditions, the separation between siRNAs and proteins used for their production might not be achieved, as the siRNA and BSA peaks are not fully resolved (compare Figures S6A and S7, Supplementary Materials). 

### 2.3. Biological Properties of AF4-Purified siRNA Swarms

#### 2.3.1. AF4 Purified siRNA Swarms Are Not Toxic for Mammalian Cells

Mammalian cells are sensitive to the impurities that arise from the enzymatic production of siRNA preparation. For instance, dsRNA molecules that are longer than 30 base pairs (bp) activate the mammalian interferon system and lead to cell death via apoptosis or necroptosis [72,73]. Therefore, it is crucial that the purification procedure for siRNAs removes all undigested or partially digested dsRNA molecules. Other contaminants, such as ssRNAs, rNTPs, and enzymes used for siRNA production, should be also avoided. To assess the quality of siRNA purification with AF4, toxicity tests were performed in vitro using two cell lines, human nervous system-derived cells U-373 MG and human corneal epithelium (HCE) cells. The cell cultures were selected to represent tissues naturally susceptible to HSV-1 infection. Swarms of siRNAs purified with AEX (Figure S8) were used as a reference since the AEX method for siRNA molecules is well established and extensively verified [31,49,62,63,74]. 

**Figure 7:**
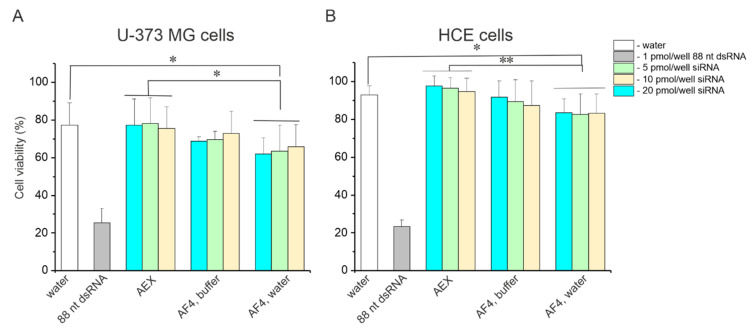
Toxicity of AF4-purified siRNA swarms. Viability of human nervous system derived U-373 MG cells (**A**) and human corneal epithelium (HCE) cells (**B**) treated with either AEX- or AF4-purified siRNA swarms. The cells were transfected with 5, 10 or 20 pmols of the purified eGFP or UL29 siRNAs per well, 1 pmol/well of 88-nt dsRNA or water. After 48 h post transfection, the cell viability was determined using CellTiter-Glo luminescent assay. The viability was calculated as a percentage from the viability of intact cells. Data are presented as the mean ± S.D. Asterisks represent a significant difference determined by Mann–Whitney U test either between a control (mock transfection) and a group of comparison or between two indicated groups; *—*p* < 0.05, and **—*p* < 0.01.

We evaluated the toxicity of siRNA batches purified with AF4 in either 20 mM NaCl, 50 mM Tris-HCl (pH 8.0), or in ultrapure water (see Section 2.2.3; Figure 5). To this end, the U-373 MG or HCE cells were transfected with the purified siRNAs on 96-well plates using 5, 10, or 20 pmols siRNA per well. Water was used for mock transfection. Transfection with 1 pmol of 88-nt dsRNA was included as a toxicity control since it leads to cell death [31]. As expected, the 88-nt dsRNAs caused about a 75% drop in the viability of U-373 MG and HCE cells, which verified the successful transfection procedure. In general, U-373 MG cells were more sensitive to transfection compared to HCE cells, and we observed about 75% and 90% cell viability in U-373 MG and HCE cells, respectively, after mock transfection (Figure 7). The viability of the cells did not differ significantly between the mock transfection control and transfection with AEX-purified siRNAs. SiRNAs separated with AF4 in a buffered mobile phase were also not toxic to the cells (Figure 7A,B). Notably, the siRNAs are typically used in amounts below 10 pmols/well to induce efficient gene knockdown in mammalian cells, but even 2-fold higher concentrations of the siRNAs were well tolerated by the cells used in the experiments. However, siRNAs purified with AF4 using ultrapure water as a mobile phase caused a slight but statistically significant drop in cell viability for both U-373 MG and HCE cells (Figure 7A,B), indicating compromised purity of these siRNA batches. 

#### 2.3.2. AF4 Purified siRNA Swarms Retain Their Antiviral Activity 

**Figure 8:**
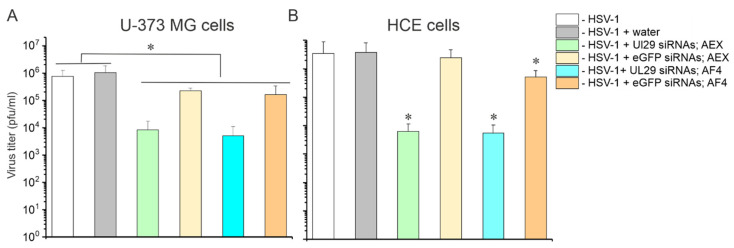
Biological properties of AF4-purified siRNA swarms. To evaluate the antiviral activity of AEX- and AF4-purified siRNA swarms, the U-373 MG (**A**) and HCE (**B**) cells were transfected on 96-well plates with 5 pmols/well of either HSV-1-specific UL29 siRNAs or control non-specific eGFP siRNAs. After 4 h post transfection, the cells were infected with 1000 plaque forming units (PFU) of HSV-1 17+ strain. After 44 h post infection, the supernatant was collected for plaque assay. Data are presented the as mean ± S.D. Asterisks represent a significant difference either between a control (water transfection or water transfection + infection) and a group of comparison or a difference between two indicated groups as determined by Mann–Whitney U test; *—*p* < 0.05.

We have previously demonstrated antiviral activity of AEX-purified UL29 siRNA swarms against HSV infection by measuring viral shedding with a plaque assay and UL29-specific mRNA expression by quantitative reverse transcription PCR [31]. To confirm that AF4 purification of siRNA swarms does not affect their antiviral properties, we transfected U-373 MG and HCE cells with 5 pmol/well of siRNAs, and four hours later we infected the cells with 1000 plaque-forming units (PFU) of HSV-1 17+ strain. At the time point of 48 h post transfection (hpt), the supernatants were collected and titrated on Vero cells for a plaque assay. According to the assay, the HSV-1 had similar titers, ~1.0 × 10^6^ PFU/mL and ~3.4 × 10^6^ PFU/mL in U-373 MG and HCE cells, respectively. Mock transfection did not affect virus growth in cell cultures. The antiviral activities of the AF4- and AEX-purified UL29 siRNA swarms were similar (Figure 8) and both reduced viral titers up to three orders of magnitude (*p* < 0.01) preventing the development of morphological changes induced by HSV-1 infection. The eGFP siRNA swarm also demonstrated some non-specific antiviral activity (Figure 8) related to the activation of innate immune responses [62,74], but this effect was not as prominent as that of HSV-specific siRNA swarm. 

## 3. Discussion

Small RNA molecules are becoming increasingly important therapeutic modalities, and new advanced methods for their purification, separation, and analysis are needed. Furthermore, newly discovered sRNA molecules need to be purified to facilitate their structural and functional studies. AF4 is a versatile separation technique, which allows gentle purification of biomolecules in their native state. We investigated the elution behavior of sRNA molecules, their potential purification and separation with the AF4 system. Furthermore, we applied AF4 coupled to a multidetector system (MALS, RI, UV, and fluorescence detector) to characterize molar mass, homogeneity, and recovery of sRNA preparations. We used two types of ssRNA and dsRNA molecules, 88- and 108-nt-long, as well as 27-nt-long siRNAs (Table 1). 

The uniform hydrodynamic size of the short dsRNA molecules and their rod-shaped structure made them more feasible purification targets compared to single-stranded molecules, which are prone to adopt alternative conformations (Table 1, Figures S1 and S2) and form multimeric complexes (Figure 2C,D, Figures S4 and S5). The pre-purified 88- and 108-nt dsRNAs eluted as single homogenous peaks (Figure 3) and a sufficient separation was achieved between the monomeric ssRNA form and the dsRNA when the crude dsRNA synthesis reaction mixtures were analyzed (Figure 3). 

The 108-nt ssRNA studied here appeared to have a tendency to form multimeric complexes, which resulted in its elution in two main peaks (Figure 2C,D). Thus, our results suggest that AF4 could be used for the detection of alternative RNA forms and studies of RNA folding and multimerization. The formation of such multimers can be probably induced during the focusing step of the AF4 experiment, where the analyte is concentrated to a small volume (approximately 1–2 µL) potentiating intermolecular interactions [57]. Such observation suggests that AF4 could provide a means to promote the detection of alternative RNA forms and to study RNA folding and multimerization. However, if the formation of such structures compromises the purification process, the sample injection technique implemented in frit-inlet/dispersion channel, which omits the focusing step, could provide a solution [57]. The composition of a mobile phase affects the elution behavior of negatively charged RNA molecules [68]. Accordingly, the mobile phase with low ionic strength substantially decreased the intermolecular interactions observed between 108-nt ssRNAs and reduced the retention time of the analytes (Figure S5). 

SiRNA molecules were efficiently separated by AF4 from partially digested dsRNAs, NTPs, and enzymes used in their production (Figure 4 and Figure 5). The method for purification of enzymatically created siRNA swarms was verified with two AF4 instruments currently available on the market; AF2000 MultiFlow FFF (Postnova Analytics) and Eclipse NEON (Wyatt). Both systems provided a similar level of siRNA purity. Despite our initial concern that 10 kDa RC membrane will not retain siRNA, the measured recovery using 10 kDa membrane was about 70% in the mobile phase containing 20 mM NaCl in 50 mM Tris-HCl (pH 8.0). This exceeds an average siRNA recovery obtained with AEX by 20% [31]. The AF4 operation using a membrane with 10 kDa MWCO instead of 1 kDa also allows higher flow rates reducing problems related to overpressure and resulting in a better separation and shorter analysis time. 

We studied if water can be used as a mobile phase for siRNA purification. The idea of this experiment arose from the presumption that AF4 separations in water might potentiate the application of siRNA swarms directly to the cells as no buffer exchange or desalting steps are required. However, when water was used as a mobile phase, siRNAs eluted in a void volume, which complicated their separation from small sample components (Figure 6 and Figure S7). This translated into a slightly but statistically significantly increased cellular toxicity of siRNAs swarms purified in plain water (Figure 7). Therefore, to accomplish efficient separation of siRNAs from impurities, a buffered solution containing 10–20 mM salt should be used. The siRNAs purified in 20 mM NaCl, 50 mM Tris-HCl (pH 8.0) were not toxic to the human cells in vitro (Figure 7) and effectively inhibited HSV-1 infection (Figure 8). 

Although AF4 was designed for the separation and characterization of large macromolecules and macromolecular complexes, we demonstrated that it is also applicable for the purification of short ssRNA and dsRNA molecules, including siRNAs. The amounts of RNA loaded into AF4 channels in this study were limited to tens of micrograms. However, large-scale purification would require scaling up of the injected RNA amounts to speed up the siRNA production process. The AF4 semi-preparative channels are currently available on market enabling upscaling from microgram to milligram amounts. Combination of the AF4 separation with MALS, RI, and UV detectors allows monitoring of the quantity of the product as well as a number of quality aspects, such as molar mass, polydispersity, radius of gyration, and length of RNA molecules.

## 4. Materials and Methods

### 4.1. Production and Purification of sRNA Molecules

#### 4.1.1. Generation of sRNA Molecules 

We generated five types of sRNAs for the study (Table 1). To produce 88- or 108-nt ss- and dsRNA, plasmid pLM659 containing a complementary DNA copy of the genomic S-segment of bacteriophage phi6 (GenBank accession number NC_003714) [75] was used as a template in PCR. The amplified DNA molecules comprise 80 nt or 100 nt from the very 3′ end of the S-segment. The primers used for template amplification contained promoter regions for T7 and φ6 polymerases bringing the extra 8 nt in the resulting RNA sequence [59]. DNA templates for the production of siRNA swarms were PCR-amplified from plasmids pET32UL29 [31] or pCR3.1-eGFP [29]. Plasmid pET32UL29 contains 653 bp sequence derived from the *UL29* gene of HSV-1 prototype strain 17+ (GenBank JN555585.1, nucleotides 59,302 to 59,954) and plasmid pCR3.1-eGFP harbors complete 717 bp *eGFP* gene subcloned from pEGFP-C1 (GeneBank U55763). In the manuscript text, these dsRNAs are referred to as 650 bp and 720 bp, respectively. Gel-purified PCR products were used as templates to generate ssRNA by T7 DNA-dependent RNA polymerase. The dsRNA molecules were produced in coupled reactions using T7 and phi6 polymerase [29]. 

After DNase treatment (RQ1 RNase-Free DNAse, Promega, Madison, WI, USA), the RNAs were directly used for AF4 injections or further purified prior to the AF4 analysis. The pre-purification of ssRNAs included TRIsure-chloroform extraction (TRIsure, Bioline GmbH, Luckenwald, Germany) and precipitation with 4 M lithium chloride (LiCl, Merck, Darmstadt, Germany), while pre-purification of dsRNA molecules included consecutive precipitations with 2 M and 4 M LiCl (Figure 1). The RNAs were stored at −80 °C. A fraction of prepared 88-nt dsRNA molecules was purified with AEX (Section 4.1.3) to serve as a reference for the cell viability analyses. 

To generate siRNA swarms, pre-purified UL29 and eGFP dsRNAs were digested into 27-nt siRNAs using the Dicer enzyme from *Giardia intestinalis* [31]. The enzyme was expressed in Bac-to-Bac baculovirus expression system and purified using HisPur Ni-NTA resin (Thermo Fisher Scientific; Rockford, IL, USA) by the facility of protein services of the University of Tampere. Half of the prepared siRNA swarms were subjected to AF4 purification, and the rest was purified using AEX as previously described [49], with modifications highlighted in 4.1.3. For preliminary AF4 experiments, we used siRNA swarm obtained after digestion of bacteriophage phi6 dsRNA genome (GenBank accession numbers NC_003714, NC 003715, NC 003716) with Dicer. To this end, bacteriophage phi6 [76] was propagated and purified as described previously [77]. Total RNA was extracted from viral particles using TRIzure reagent (Bioline GmbH, Luckenwald, Germany) and chloroform according to the manufacturer’s instructions. The dsRNA was fractioned using LiCl precipitation and cleaved with Giardia Dicer into siRNA swarms.

#### 4.1.2. AF4 Setup and Operation

We performed the initial AF4 experiments and data collection with the set-up previously described [78] using the AF2000 MT instrument (Figure 2, Figure 3, Figure 4, Figure 6 and Figure S3) and Postnova AF2000 control software, version 2.0.1.5 (Postnova Analytics, Landsberg, Germany). The injection loop volume was 100 µL unless mentioned otherwise. The volume of the analyzed sample was adjusted to 110 µL with the used mobile phase (see below). For the pre-purified samples, this corresponded to ~20–35 µg of RNA. For the crude samples, the appropriate injection volume was selected by performing pre-analyses with different sample volumes. A UV detector equipped with an analytic flow cell (Shimadzu SPD-20A; Shimadzu, Kyoto, Japan) was used to monitor the channel outlet flow signal in volts (V) at 260 nm. The AF4 program consisted of four steps: (1) injection with a 5 min focusing step; (2) initial elution at constant cross-flow (V_c_) of 2.3 mL/min for 20 min; (3) V_c_ linearly decaying to 0.1 mL/min in 5 min, and (4) constant V_c_ of 0.1 mL/min for 15 min. Channel outlet flow (V_out_) was 0.3 mL/min. Injection flow was 0.3 mL/min. Fractions of 0.3 (1 min) or 0.6 mL (2 min) were collected during the elution step. Retention times (t_r_) with focusing and transition time deducted were obtained from the peak maxima. Alternatively, the Eclipse NEON FFF system (Wyatt Technology, Santa Barbara, CA, USA) comprising an Agilent 1260 Infinity II pump and autosampler, analytical long channel, the Eclipse FFF flow instrument, dilution control module (DCM), Agilent 1260 fraction collector, was used for AF4 purification of siRNA molecules (Figure 5, Figures S3, S4, S6, and S7). The Eclipse NEON FFF system was connected to DAWN, a Multi-Angle Light Scattering detector with 18 angles (MALS, Wyatt Technology, Santa Barbara, CA, USA), one of which is occupied by WyattQELS online dynamic light scattering (DLS) detector, Agilent 1260 Infinity II UV and fluorescence detectors as well as Optilab differential refractometer (dRI) detector (Wyatt Technology, Santa Barbara, CA, USA). A UV detector monitored the nucleic acid signal at 260 nm, whereas a fluorescence detector was utilized to monitor proteins (excitation at 280 nm, emission at 340 nm) The FFF-MALS was controlled via VISION 3.0.0.20 software (Wyatt Technology, Santa Barbara, CA, USA). Data were analyzed with ASTRA 8.0.0 software (Wyatt Technology, Santa Barbara, CA, USA). The optimized flow program for the ECLIPSE NEON is shown in Table S1. The channel flow was 1 mL/min, inject flow 0.2 mL/min, and detector flow 0.5 mL/min.

A Teflon spacer adjusted the FFF separation channel to a nominal width of 350 μm (for Postnova AF2000MT instrument) or 400 µm (for the Eclipse NEON system). The MWCO of the RC membrane was 1 kDa (Postnova) or 10 kDa (Wyatt Technology). 

Unless otherwise stated, we performed AF4 experiments at 22 °C using 20 mM NaCl, 50 mM Tris-HCl (pH 8.0) buffer. Alternatively, we used 10 mM Tris-HCl (pH 8.0), or nuclease-free ultrapure water purified with the Milli-Q^®^ Direct 8 system (Merck, Darmstadt, Germany) as a mobile phase. Each fractionation was repeated at least two times. The instrument was rinsed with the used mobile phase intensively between different RNA samples.

#### 4.1.3. RNA Purification Using AEX

Chromatographic experiments were performed at room temperature using the ÄKTAPurifier 10UPC system (GE Healthcare, Uppsala, Sweden) controlled by Unicorn 5.2 software (GE Healthcare, Uppsala, Sweden). An anion exchange CIM QA-1 monolithic column (BIA Separations, Ajdovscina, Slovenia) was used for the reference RNA purification. The column has an average pore size of 1.3 µm, outer diameter of 18.6 mm, inner diameter of 6.7 mm, length of 4.2 mm, and a bed volume of 1.0 mL. Its stationary phase is based on poly(glycidylmethacrylate-co-ethylenedimethacrylate) functionalized with quaternary amines. Buffer A containing 20 mM NaCl, 50 mM Tris-HCl (pH 8.0) and buffer B containing 1 M NaCl, 50 mM Tris-HCl (pH 8.0) were used in AEX. Moreover, 88-nt dsRNA was purified as described previously [49]. The generated siRNAs were diluted 3.5-times with nuclease-free ultrapure water and centrifuged at 11,000× *g* for 10 min before the injection. The injection volume was 5 mL. A linear gradient of 60 column volumes from 20% to 80% buffer B at 1 mL/min was applied (Figure S8). The column was cleaned with 1 M NaOH solution between samples. Absorbance at 260 nm was monitored and 0.5 mL fractions from the peaks were collected.

#### 4.1.4. Analysis of Recovery, Quality and Quantity

Nucleic acids were recovered from the collected fractions by overnight precipitation at −20 °C in 0.3 M sodium acetate (pH 6.5) and 67% ethanol, followed by centrifugation at 10,000× *g* for 30 min. The RNA pellets were washed with 70% ethanol, air-dried, and dissolved in 10 µL of sterile nuclease-free water. For functional experiments, the fractions containing maximum amounts of siRNAs were combined and, unless purified in ultrapure water, they were desalted using illustra NAP-25 columns (GE Healthcare, Buckinghamshire, UK) followed by concentrating with the SpeedVac vacuum concentrator (Savant Instruments Inc, Famingdale, NY, USA). Nucleic acid concentrations were measured using NanoDrop2000c Spectrophotometer (Thermo Scientific). Recovery of RNA molecules was calculated with the following formula ([eluted RNA, μg]/[input RNA, μg]) × 100%. Alternatively, an algorithm from Wyatt Technology implemented in ASTRA 8 software enabled the mass recovery calculation of RNA molecules based on the eluted RNA concentration determined by dRI detector, assuming that the refractive index increment (dn/dc) for RNA is 0.17 mL/g. 

We separated nucleic acids in 1–4% (*w*/*v*) agarose gels (MetaPhor or Nusieve CTG agarose, Lonza Bioscience) prepared in Tris-borate-EDTA (TBE) buffer. Gels stained with ethidium bromide were visualized with ChemiDoc (Bio-Rad, Hercules, CA, USA). DNA ladders and markers (#SM037; #SM0331; and #SM1401, Thermo Scientific) were applied as approximate size indicators for dsRNA molecules. 

#### 4.1.5. RNase A Treatment

The fractions collected after AF4 separation of 108-nt ssRNAs were precipitated overnight at −20 °C, as described in Section 4.1.4. Then, 1 µg of RNA from peak 1 or peak 2 was incubated with 0.1 µg of RNase A (Fermentas, Vilnius, Lithuania) either in 1 × SSC buffer (3 M sodium chloride, 0.3 M sodium citrate [pH 7.0]) or in 0.1 × SSC buffer for 15 min at room temperature. The former treatment in a high salt buffer promotes only ssRNA digestions, and the latter one, occurring in low salt buffer, results in the digestion of both ss- and dsRNA species. Enzymatically produced 500-nt dsRNA [59] was used as a control.

### 4.2. Functional Tests for AF4-Purified siRNA Molecules

#### 4.2.1. Cells and Virus

A human glioma cell line U-373 MG (currently re-classified as U-251) was originally obtained from ATCC (Manassas, VA, USA). Immortalized HCE were from Prof. Arto Urtti (University of Helsinki/University of Eastern Finland, Finland). Both cell lines are susceptible and permissive to HSV-1 and suitable for antiviral siRNA treatment [31,79]. The cells were maintained in high-glucose DMEM (Gibco) with 10% (*v*/*v*) fetal bovine serum (FBS, Serana, Silicone Valley, CA, USA) and 2 mM L-glutamine (Sigma, Saint Louis, MO, USA) in 37 °C, 5% CO_2_. African green monkey kidney cells Vero (ATCC), used for plaque assays, were grown in DMEM with 5% FBS (*v*/*v*). 

The prototype HSV-1 17+ laboratory strain was used for antiviral studies and propagated as previously described [31]. 

#### 4.2.2. Transfection

The cells were propagated on 96-well plates until their confluency reached about 50% for HCE and 70% for U-373 MG cells. The cells were transfected with 5, 10, or 20 pmol of siRNA preparation per well, 1 pmol per well of 88-nt dsRNA [49], or water (mock transfection) using Lipofectamine RNAiMAX (Invitrogen, Carlsbad, CA, USA) according to the manufacturer’s forward transfection protocol. Altogether, 15 independently produced and purified batches of siRNA swarms were used for transfection experiments; the seven batches were purified with AEX to serve as a reference, three batches were purified with AF4 using the standard mobile phase [20 mM NaCl, Tris-HCl (pH 8.0)], and five batches were purified using ultrapure water as a mobile phase. All experiments were repeated two or three times with three technical replicates each. 

#### 4.2.3. Cell Viability Assay

Possible cytotoxic effects of siRNA swarms purified with AF4 were assessed 48 hpt with the CellTiter-Glo Luminescent Assay (Promega, Madison, WI, USA) according to the manufacturer’s instructions, and luminescence was quantified with VICTOR Nivo Multimode Plate Reader (Perkin Elmer, Waltham, MA, USA). The luminescent signal from untreated cells was set as 100%, and all the other values were normalized to it. Viability similar to mock-transfected cells was considered acceptable. We measured a signal from three technical replicates for each treatment, and the average of these was used in further statistical analyses (Section 4.2.5). 

#### 4.2.4. HSV-1 Infection and Plaque Assay 

After 4 hpt with 5 pmol/well of either UL29 or eGFP siRNA swarms, the U-373MG or HCE cells were washed with DMEM containing 2% FBS, infected with 1000 PFU/well of HSV-1 17+ in 100 µL DMEM with 2% FBS, and incubated 1.5 h at 35 °C on a shaker. Then, the cells were washed and supplemented with DMEM containing 7% FBS. Live-cell imaging to confirm cytopathic effects caused by HSV-1 was performed 44 h post infection using EVOS Auto FL (Thermo Fisher Scientific). Then, the culture supernatant samples were collected, and their 10-fold dilutions were used to infect monolayers of Vero cells for 1.5 h at 35 °C in 100 µL of DMEM containing 5% FBS. Then, an equal amount of DMEM with 5% FBS and 80 µg/mL of human IgG (Kiovig, Baxalta US Inc., MA, USA) was added. After 3 or 4 days post infection, the cells were fixed with methanol followed by staining with crystal violet and quantification of plaques. 

#### 4.2.5. Statistical Analysis

Statistical analysis was conducted with SPSS Statistics version 25 (IBM, Armonk, NY, USA). Statistical significances were calculated with Mann–Whitney’s non-parametric U test comparing two individual groups. 

## 5. Conclusions

We introduce a novel AF4-based method for the purification and analysis of sRNA molecules under native conditions. We found that AF4 enables rapid purification of enzymatically produced antiviral siRNAs from partially digested long dsRNA. In addition, AF4 separates monomeric single-stranded and double-stranded RNA molecules of the same length and promotes the identification of different multimeric forms of ssRNA. Recovery of AF4-purified sRNA was high, about 70% and above. AF4-purified siRNAs were not toxic for mammalian cells and AF4-purified siRNA swarm derived from the *UL29* gene sequence of herpes simplex virus 1 was biologically active as it efficiently inhibited herpes virus replication in cell cultures.

## Figures and Tables

**Table 1:** Properties of the RNA molecules used in this study.

Length (nt)	Type	Mw (kDa) ^1^	GC (%)	Number of Predicted Secondary Structures ^2^	Base Paired Regions in the Predicted Structure	Gibbs Energy Range (kcal/mol)	Predicted Length (nm) ^3^	Predicted R_g_ (nm) ^4^
27	siRNA	17.3		1	25		~7	
88	ssRNA	31.9	50.5	2	18–20	−43.4–(−)42.3		~2.6–2.7
88	dsRNA	63.8		1	88		~26	
108	ssRNA	35.7	50.9	8	15–33	−52.1–(−)49.6		~2.4–3.1
108	dsRNA	71.8		1	108		~31	

^1^ Molecular weight (Mw) was calculated from the sequence for the ssRNA molecule in the sense orientation. For dsRNA molecules, Mw is the sum of the molecular weights of the sense and antisense strands. For siRNAs, Mw is obtained by multiplication of an average molecular weight of a base pair (640 g/mol) by the number of base pairs (27 nt). ^2^ Number of folds obtained from Mfold prediction at 22 °C. For dsRNA molecules, a single rod-like fold was expected. ^3^ Length of dsRNA molecules was calculated by assuming a mean rise per base pair of 0.29 nm [64]. ^4^ Root mean square radius of gyration was calculated with the following formula: R_g_ = ~5.6 × N^0.33^, where N is the number of bases (N < 300) [65].

## Data Availability

Data is contained within the article and supplementary material.

## Supplementary Materials

The following supporting information can be downloaded at: https://www.mdpi.com/article/10.3390/ph15020261/s1, Figure S1: Predicted secondary structures for the 88-nt ssRNA at 22 °C, Figure S2: Predicted secondary structures for the 108-nt ssRNA at 22 °C, Figure S3: Molar mass distribution of the 88-nt and 108-nt RNA sample components; Figure S4: Nature of 108-nt ssRNA species fractionated with AF4; Figure S5: Impact of mobile phase composition on 108-nt ssRNA elution; Figure S6: AF4 separation of siRNA molecules from BSA and NTPs; Figure S7: AF4 separation of siRNA molecules from BSA in water; Figure S8: Anion exchange chromatography of siRNA swarms; Table S1: Method for AF4 separation used with the Postnova AF4 system; Table S2: Method for SF4 separation used with the Eclipse NEON AF4 system.
