# Supplementary material for: Native RNA Purification Method for Small RNA Molecules Based on Asymmetrical Flow Field-Flow Fractionation
Source: Pharmaceuticals (Basel). 2022 Feb 21;15(2):261. doi: 10.3390/ph15020261 (PMC8876226; doi:10.3390/ph15020261)
Supplement: Supplementary file 1 [file pharmaceuticals-15-00261-s001.zip › pharmaceuticals-1589464-supplementary.pdf]

Native RNA purification method for small RNA molecules based on asymmetrical flow field-flow fractionation

Alesia A. Levanova[1,a], Mirka Lampi[1,a], Kiira Kalke[2], Veijo Hukkanen[2], Minna M. Poranen[1,*], Katri Eskelin [1,*]

[1]Molecular and Integrative Biosciences Research Programme, Biological and Environmental Sciences, University of Helsinki, Viikinkaari 9, FI-00014, Helsinki, Finland.; alesia.levanova@helsinki.fi (A.A.L.); mirka.lampi@helsinki.fi (M.L.)

[2]Institute of Biomedicine, University of Turku, Turku, Finland; kiira.m.kalke@utu.fi (K.K.); veijo.hukkanen@utu.fi (V.H.)

[a]Both authors contributed equally

[*]Correspondence: katri.eskelin@helsinki.fi (K.E.); minna.poranen@helsinki.fi (M.M.P.)

Content of pages 1-9

Figure S1. Predicted secondary structures for the 88-nt ssRNA at 22°C.

Figure S2. Predicted secondary structures for the 108-nt ssRNA at 22°C.

Figure S3. Molar mass distribution of the 88-nt and 108-nt RNA sample components.

Figure S4. Nature of 108-nt ssRNA species fractionated with AF4.

Figure S5. Impact of mobile phase composition on 108-nt ssRNA elution.

Figure S6. AF4 separation of siRNA molecules from BSA and NTPs.

Figure S7. AF4 separation of siRNA molecules from BSA in water.

Figure S8. Anion exchange chromatography of siRNA swarms.

Table S1: Method for AF4 separation used with the Postnova AF4 system.

Table S2: Method for AF4 separation used with the Eclipse NEON AF4 system.

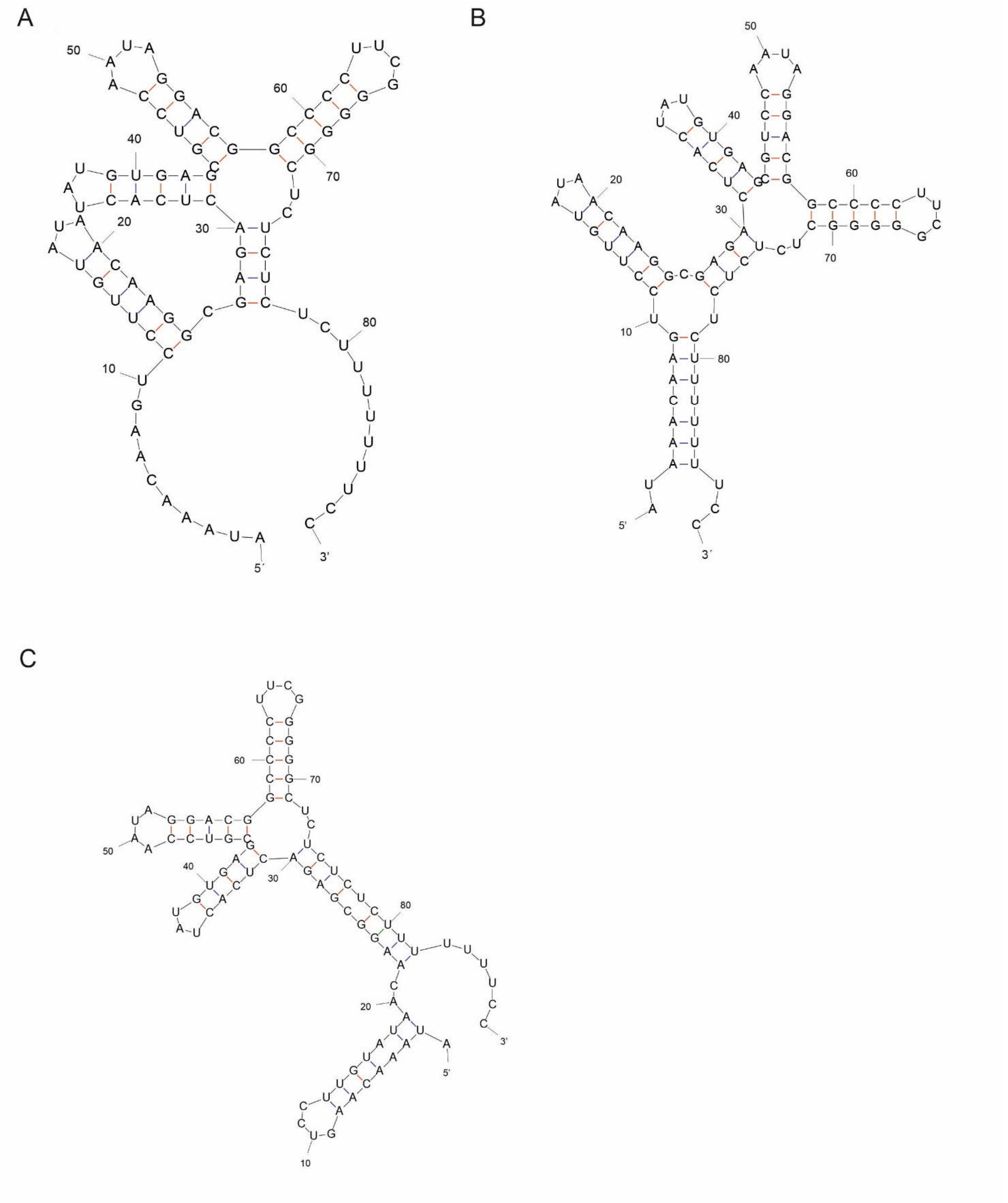

Figure S1. Predicted secondary structures for the 88-nt ssRNA at 22°C. A) ΔG -40.0 kcal/mol, B) ΔG -39.8 kcal/mol, C) ΔG -38.9 kcal/mol. Structures were predicted with MFOLD version 2.3.

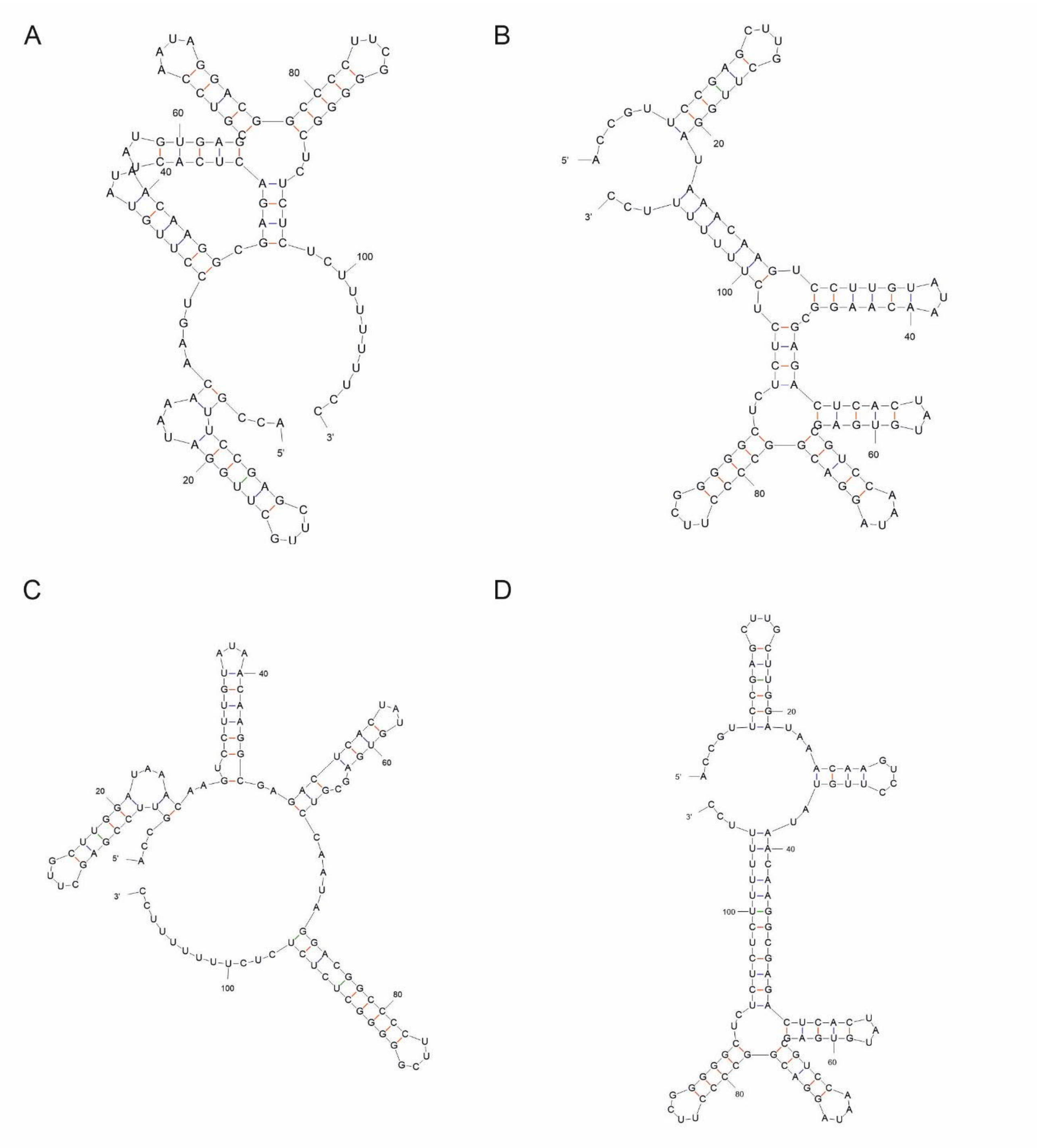

Figure S2. Predicted secondary structures for the 108-nt ssRNA at 22°C. A) ∆G -50.3 kcal/mol, B) ∆G -49.4 kcal/mol, C) ∆G -48.1 kcal/mol, D) -48.0 kcal/mol. Structures were predicted with MFOLD version 2.3.

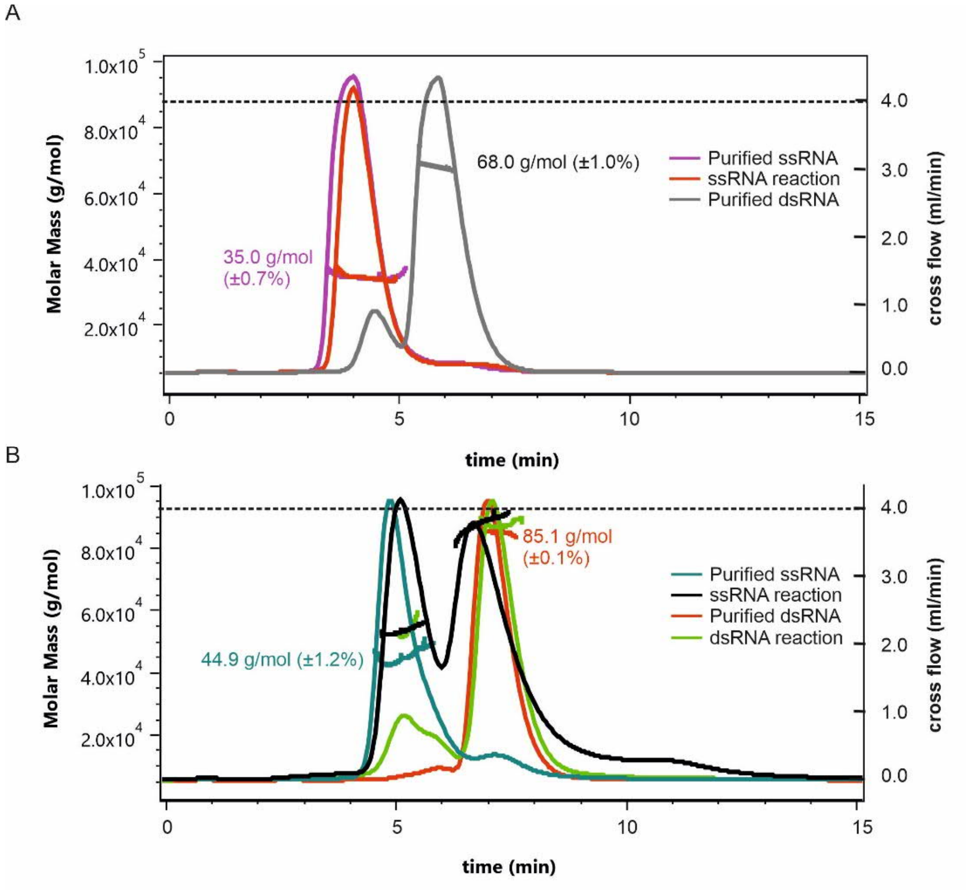

Figure S3. Molar mass distribution of the 88-nt and 108-nt RNA sample components. Representative AF4 chromatograms from the analysis of A) pre-purified 88-nt ssRNA and dsRNA as well as 88-nt ssRNA reaction, and B) pre-purified 108-nt ssRNA and dsRNA as well as corresponding reactions. The AF4 fractionation was done in 20 mM NaCl, 50 mM Tris-HCl (pH 8.0). Molar mass estimates (left y-axis) for the ssRNA and dsRNA species are indicated. Cross-flow velocity is shown with dashed line on the right y-axis. Chromatograms show UV-signal on a relative scale. Channel flow was 1 ml/min and detector flow 0.5 ml/min. Analysis was performed with NEON Eclipse instrument.

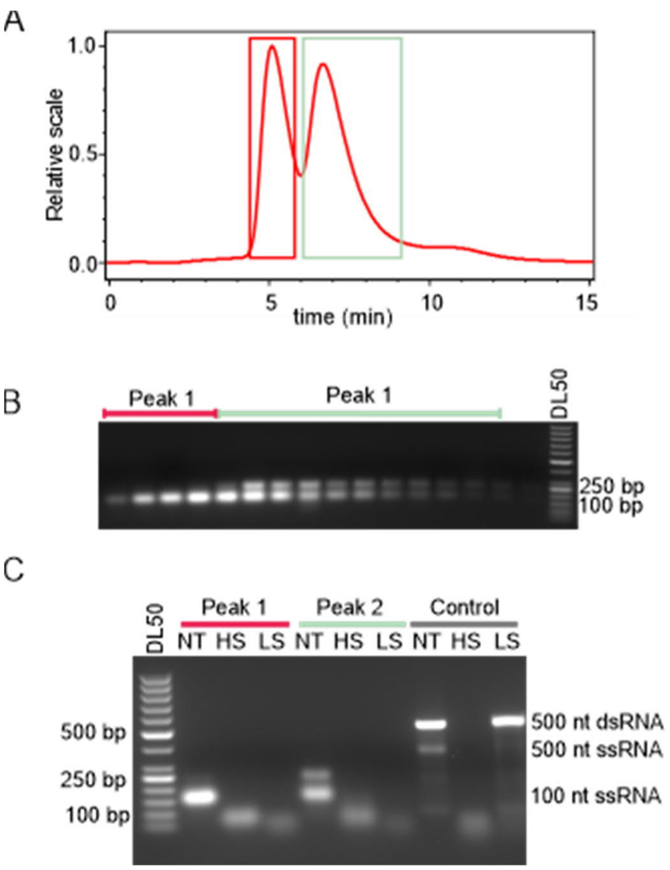

Figure S4. Nature of 108-nt ssRNA species fractionated with AF4. A) Representative AF4 chromatogram for the pre-purified 108-nt ssRNA eluted in 20 mM NaCl, 50 mM Tris-HCl (pH 8.0). B) Native agarose gel electrophoresis [1.5% (w/v) agarose] of the fractions collected from the peak 1 and 2 (boxes in red and mint green, respectively). C) RNaseA treatment of the combined fractions from peak 1 or peak 2 was performed in high-salt buffer (HS) which results in the digestion of ssRNAs only and in low salt buffer (LS) which induces degradation of both ss- and dsRNA species. Enzymatically produced 500-nt dsRNA that contained ssRNA as impurity was used as a control. Agarose gel electrophoresis of the non-treated samples (NT) and RNase treated samples (HS and LS conditions) is shown for both peaks. DL50 is GeneRuler 50 bp DNA ladder. AF4 fractionation was performed with NEON Eclipse instrument. Cross-flow velocity was 4 ml/min, channel flow 1 ml/min and detector flow 0.5 ml/min, respectively. UV signal is shown with relative scale.

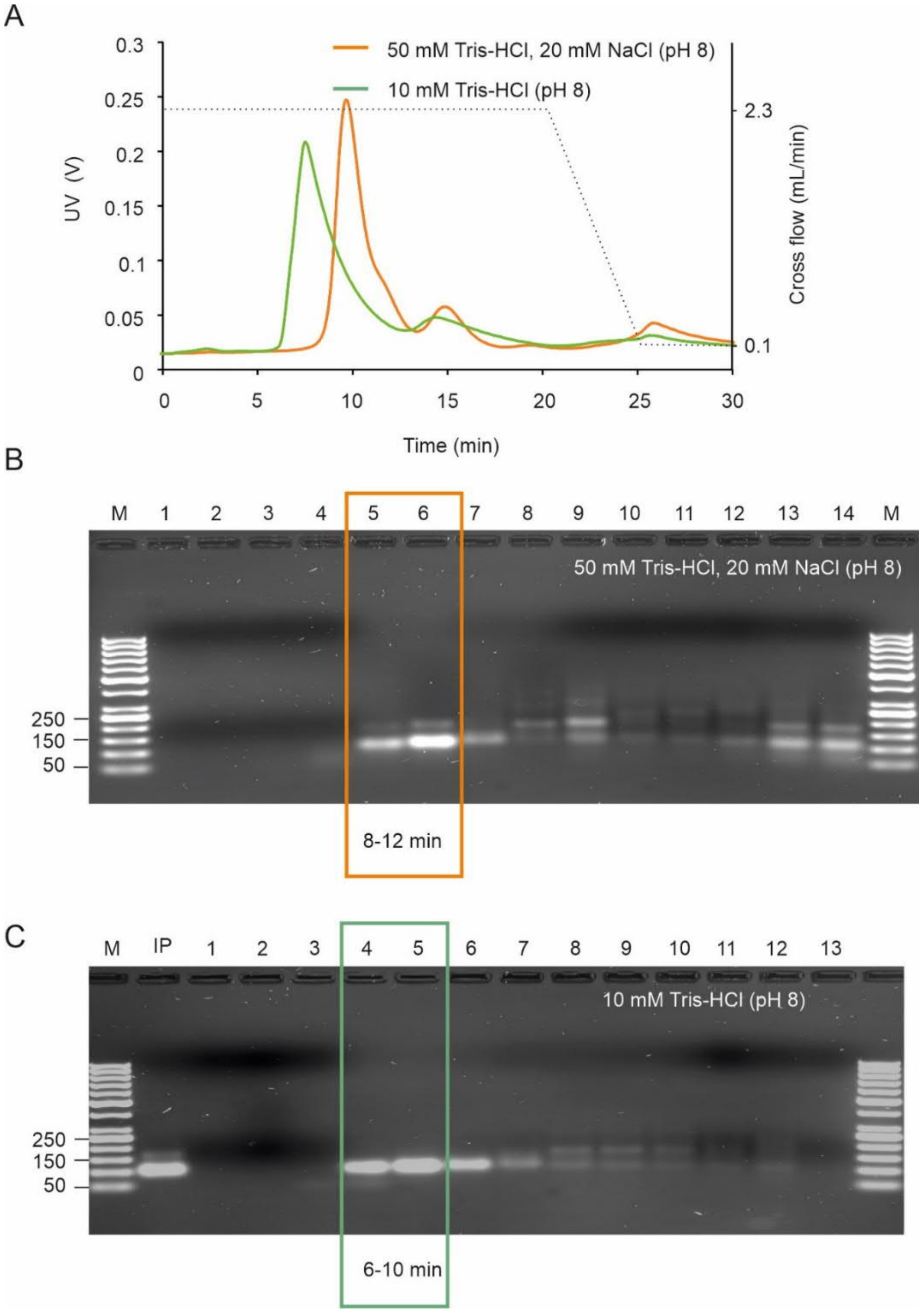

Figure S5. Impact of mobile phase composition on 108-nt ssRNA elution. A) Representative fractograms from the analysis of the pre-purified 108-nt ssRNA in the presence of 50 mM Tris-HCl (pH 8.0) supplemented with 20 mM NaCl (orange), or 10 mM Tris-HCl (pH 8.0) without NaCl supplement (green). Cross-flow gradient is shown with dashed line (right y-axis). Time axis shows the UV fractogram from the beginning of elution program excluding the focusing time. UV detector response is given at 260 nm in volts (V) (left y-axis). B-C) Native agarose gel electrophoresis [1% (w/v) agarose] of the peak, pre-peak and post-peak fractions from AF4 fractionation done B) in the presence of 20 mM NaCl or C) without NaCl. Peak position is indicated with a box. The analysis was performed with Postnova AF2000 MT instrument. The 108-nt ssRNA input sample (IP) is shown in C). M is GeneRuler 50 bp DNA ladder (Thermo Fisher).

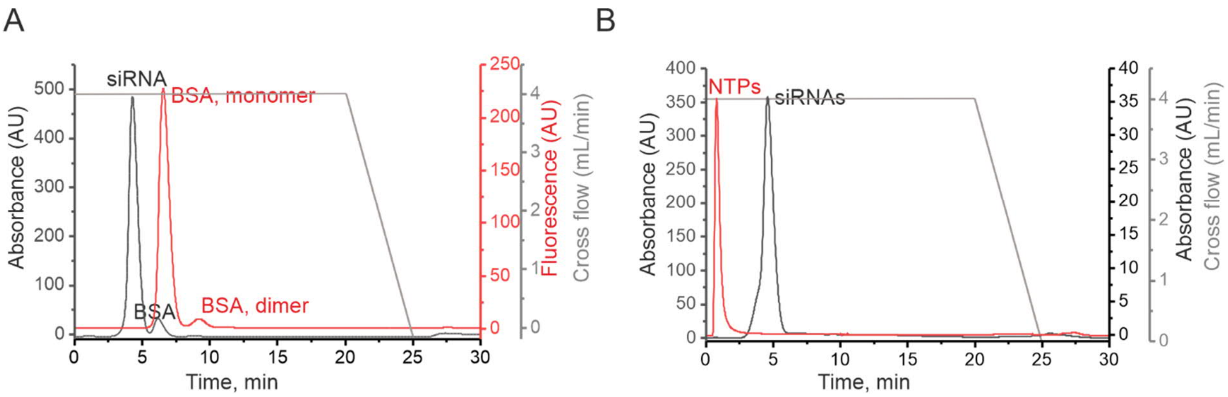

Figure S6. AF4 separation of siRNA molecules from BSA and NTPs. A) A 10 µg sample of siRNA pre-purified with AEX was combined/mixed with 30 µg of BSA and injected to the AF4 instrument. UV detector response at 260 nm for siRNA monitoring is shown in arbitrary units (left y-axis). Fluorescence detector (excitation at 280 nm, emission at 340 nm) was used for BSA detection (right y-axis). BSA monomer and dimer are well detected with fluorescence detection (red), whereas signal intensity at 260 nm is low. B) A sample containing 225 µg of NTPs was injected to the AF4 instrument. For comparison, a fractogram of siRNA elution is shown in the same graph. UV detector response at 260 nm is shown in arbitrary units (left y-axis for siRNA and right y-axis – for NTPs). Eclipse NEON FFF system (Wyatt) was used for the separation of the mixtures with the program presented in Table S1. Standard mobile phase, comprising 20 mM NaCl, 50 mM Tris-HCl pH 8.0 was used.

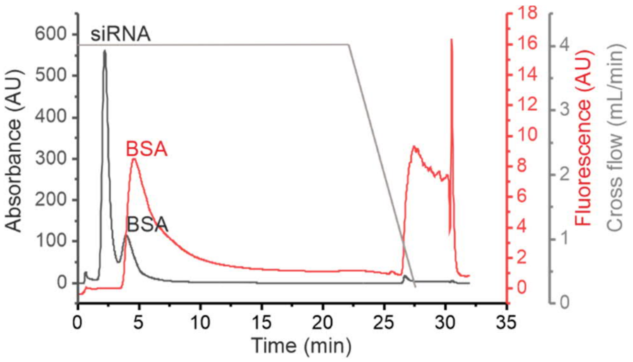

Figure S7. AF4 separation of siRNA molecules from BSA in water. 15 µg of AEX purified siRNAs (17.7 kDa), obtained after Giardia Dicer digestion of phi6 dsRNA genome, was mixed with BSA (66.5 kDa). Eclipse NEON FFF system (Wyatt) was used for the separation of the mixture with the program presented in Table S1. An ultrapure water was used as a mobile phase. Representative fractogram for the separation of siRNA swarm (black line) from BSA (red line). UV detector response at 260 nm for siRNA monitoring is shown in arbitrary units (AU, left y-axis). Fluorescence detector was used for BSA detection (right y-axis).

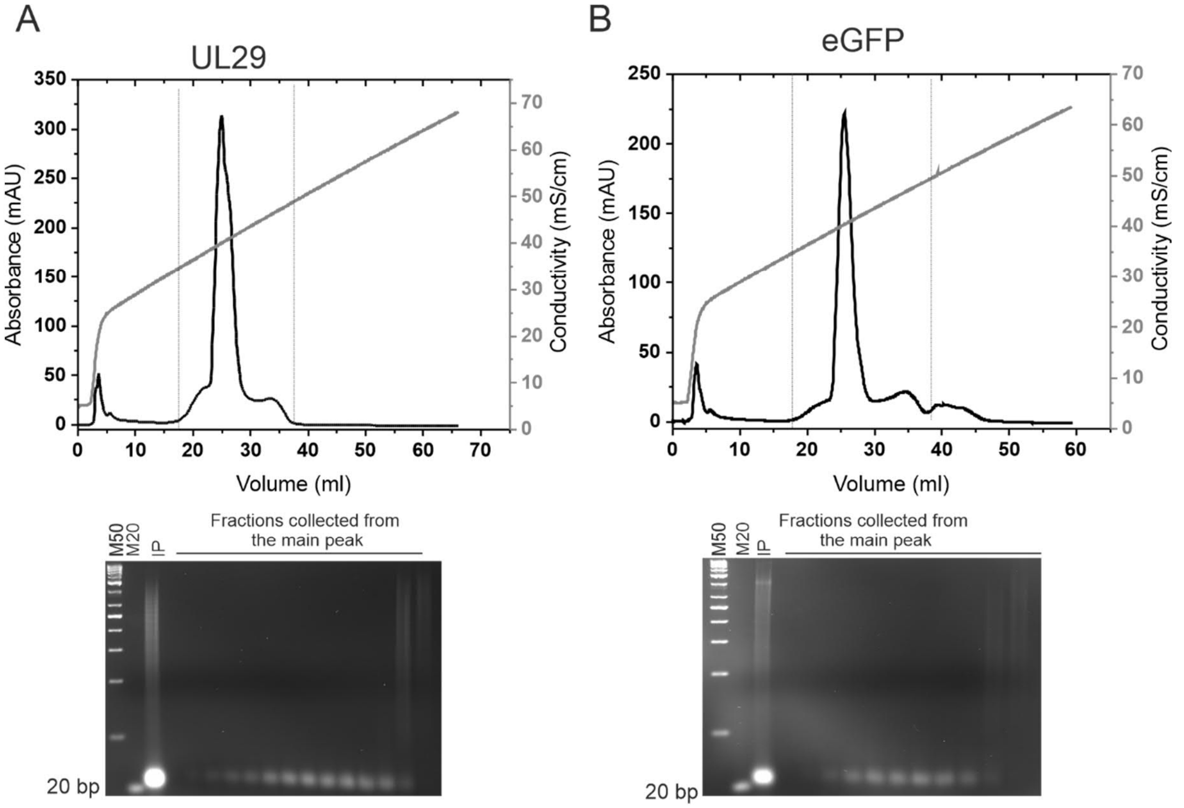

Figure S8. Anion exchange chromatography of siRNA swarms. Enzymatically produced dsRNAs (130 µg) were cleaved with Giardia Dicer and obtained UL29 siRNAs (A) and eGFP siRNAs (B) were loaded onto a CIM QA-1 monolithic column equilibrated with 20 mM NaCl, 50 mM Tris-HCl (pH 8.0) (buffer A). A linear gradient from 20% to 80% of the 1 M NaCl, 50 mM Tris-HCl (pH 8.0) (buffer B) was applied. Fractions of 0.5 ml from the main peak (indicated with grey lines) were collected and analyzed in 4% native agarose gel. The middle fractions containing siRNAs were combined and desalted before further use. GeneRuler 50 bp DNA ladder (M50) and 20 bp dsDNA marker (M20) were used to verify the size of purified siRNAs. The elution of siRNA was monitored at 260 nm with UV detector and the absorbance (mAU) is plotted against the elution volume. The buffer conductivity (grey line) is shown on the right y-axis.

Table S1: Method for AF4 separation used with the Postnova AF4 system. Channel and detector flow velocities were 0.3 ml/min.

Mode	Duration, min	Cross flow, start, ml/min	Cross flow, end, ml/min	Flow profile
Focus-Inject	5	2.3	2.3	constant
Elution	20	2.3	2.3	constant
Elution	5	2.3	0.1	linear
Elution	5	0	0	constant

Table S2: Method for AF4 separation used with the Eclipse NEON AF4 system. Channel flow velocity was 0.6 ml/min and detector flow velocity 0.5 ml/min.

Mode	Duration, min	Cross flow, start, ml/min	Cross flow, end, ml/min	Flow profile
Elution	2	4	4	constant
Focus	1	3.5	3.5	constant
Focus-Inject	3	3.5	3.5	constant
Focus	5	3.5	3.5	constant
Elution	20	4	4	constant
Elution	5	4	0.1	linear
Elution-Inject	5	0	0	constant
Elution	2	4	4	constant
